# Opioid use after total hip arthroplasty surgery is associated with revision surgery

**DOI:** 10.1186/s12891-016-0970-6

**Published:** 2016-03-10

**Authors:** Maria C. S. Inacio, Nicole L. Pratt, Elizabeth E. Roughead, Elizabeth W. Paxton, Stephen E. Graves

**Affiliations:** Medicine and Device Surveillance Centre of Research Excellence, Sansom Institute, School of Pharmacy and Medical Sciences, University of South Australia, GPO Box 2471, Adelaide, 5001 South Australia Australia; Quality Use of Medicines and Pharmacy Research Centre, Sansom Institute, School of Pharmacy and Medical Sciences, University of South Australia, GPO Box 2471, Adelaide, 5001 South Australia Australia; Surgical Outcomes and Analysis, SCPMG Clinical Analysis, Kaiser Permanente, 8954 Rio San Diego Drive, Suite 406, San Diego, CA 92108 USA; Australian Orthopaedic Association National Total Joint Replacement Registry, South Australian Health and Medical Research Institute, Adelaide, South Australia Australia

**Keywords:** Opioids, Total hip arthroplasty, Revision, Analgesic drugs

## Abstract

**Background:**

Pain is an indication for total hip arthroplasty (THA) and it should be resolved post-surgery. Because patients’ pain is typically treated pharmacologically we tested whether opioid use can be used as a surrogate for patient-reported pain and as an indicator for early surgical failure. Specifically, we evaluated whether the amount of opioids taken within the year after THA was associated with one and five years risk of revision surgery.

**Methods:**

A cohort of 9943 THAs (01/2001-12/2012) was evaluated. Post-operative opioid use was the exposure of interest and cumulative daily oral morphine equivalent (OME) amounts were calculated. Total OMEs/90-day periods were categorised into quartiles. Revisions within one and five years were the outcomes of interest.

**Results:**

Of the THAs, 2.0 % (*N* = 200) were revised within one year and 4.2 % (*N* = 413) within five years. After adjustments for gender, age, surgical indication, co-morbidities, and other analgesics, revision was associated with amount of OMEs in the second quarter after THA (days 91–180 after discharge). Patients on medium-high amounts of OME (400-1119 mg) had higher risk of one (hazard ratio (HR) = 2.22, 95 % CI 1.08-4.56) and five year (HR = 1.66, 95 % CI 1.08-2.56) revision than a patient not taking opioids. During the same period, patients taking the highest amounts of OMEs (≥1120 mg) had a 2.64 (95 % CI 1.03-6.74) times higher risk of one year and a 2.11 (95 % CI 1.13-3.96) times higher risk of five year revision.

**Conclusions:**

Opioid use 91–180 days post-surgery is associated with higher risk of revision surgery and therefore is an early and useful indicator for surgical failure.

## Background

Pain is one of the main indications for total hip arthroplasty (THA) and presence of pain post-operatively is an important indicator of procedure’s success [1, 2]. When pain is still present post-operatively, and is not related to another condition, it is typically the sign of a surgical complication or a non-successful outcome. Post-operative chronic pain has been estimated to occur in 7 % to 23 % of THA patients [3]. Additionally, 4 % of primary THAs are revised due to pain alone as post-operative pain is associated with revision THA [4, 5].

Typically, musculoskeletal pain is assessed using patient reported outcome tools [6]. While patient reported outcomes tools can be useful in certain settings, the instruments used to measure patient reported outcomes have varying validity and reliability, and can be challenging to implement because of cost, and difficulty integrating with workflows and gaining patient participation [2, 7]. Opioids are recommended for moderate to severe musculoskeletal pain after other pharmacological analgesic interventions have failed, are contra-indicated, or when refractory pain is present [8–10]. Because patients’ pain is usually treated pharmacologically, and opioids are only recommended and should therefore only be prescribed to a patient when enough pain is present, it is possible this could be used as a surrogate for patient reported pain assessment. Therefore, pharmaceutical data offers a potentially efficient and economical approach for assessing pain, which can be implemented as part of the surveillance for large cohorts of patients for early signals of potential surgical success.

In order to determine whether pain medication use could be used as an indicator for THA success, we evaluated the prevalence of pain medication use in patients after THA surgery and its association with revision surgery. Specifically, we evaluated whether the amount of opioids taken over the year after a primary THA procedure was associated with the one year and five years risk of revision after adjusting for other analgesic use, patient demographics, co-morbidities, and indications for surgery.

## Methods

### Study design, setting, and sample

A retrospective cohort study of Australian patients undergoing THA from 01/01/2001 to 31/12/2012 was undertaken using data from the Department of Veterans Affairs (DVA) administrative health claims database. In the dataset, medications are coded according to the World Health Organisation Anatomic, Therapeutic and Chemical classification (ATC), and the Australian Pharmaceutical Benefits Schedule (PBS) item codes [11, 12]. Hospitalisations are coded according to the International Classification of Diseases, 10^th^ Revision, Australian Modification (ICD-10-AM) [13] scheme.

The study sample included patients aged ≥18 years old that underwent primary, elective, unilateral THA procedures (identified using ICD-10-AM code 4931800). Patients with multiple arthroplasties (inclusive of knee, resurfacing procedures, or hip fractures) within 365 days of the index THA were not included. Additionally, any patients with history of malignancy or lymphoma (ICD-10-AM C00.x–C26.x, C30.x–C34.x, C37.x–C41.x, C43.x, C45.x–C58.x, C60.x–C76.x, C81.x–C85.x, C88.x,C90.x–C97.x, C77.x–C80.x, C81.x–C85.x, C88.x, C96.x, C90.0, C90.2) or dispensed antineoplastic and immunomodulating agents within their medication history (ATC codes L01*) were not included. The obtained sample had 10,018 patients, of which 75 (0.7 %) were excluded as they were considered outliers with their opioid use being in the 99^th^ percentile (i.e. > 7350 mg of oral morphine equivalents (OME) in days 0–90, >9860 OME mg in days 91–180, >11088 OME mg in days 181–270, and > 9840 OME mg in days 271–260) of the total use. The final study sample had 9943 patients.

### Outcomes of interest

The main outcome of interest was revision within one year and five years. Using the hospitalisation dataset, the following ICD-10-AM procedure codes were used to identify revisions: 4932100, 4932400, 4932700, 4933000, 4933300, 4933900, 4934200, 4934500, and 4934600.

### Exposure of interest

The cumulative daily amount, calculated as OME, of oral and transdermal opioid medication (ATC code N02A*), was calculated over the 360 days post discharge from the THA procedure [14]. OMEs were calculated using a standard conversion table to translate the dose and type of each opioid a patient received into one morphine equivalent dose [14] for overall comparison of analgesic dose. Total post-THA oral morphine equivalents per 90 day exposure periods categorised into quartiles were the exposure of interest of this study. If a patient had a revision or died during the 360 day post-operative period, the amount of opioid exposure was calculated until the day before the event occurred. The cumulative OME daily dose was calculated by summing the total dose per day based on quantity of supply. The total OME for a 90 day exposure time was the sum of all OME daily doses for that period.

### Covariates/confounders

Patient gender, age, primary diagnosis, co-morbidities, as well as prior opioid use, and pre- and post-operative non-steroidal anti-inflammatory drug (NSAID) use were evaluated as possible confounders. Counts of comorbidity were made using a modified RxRisk-V co-morbidity measure, which identified 42 conditions [15]. In an attempt to further control for the possibility of confounding due to back pain (not specifically identified in the RxRisk-V), we used a previously published Australian back pain ICD-10-AM coding algorithm to identify patients with history or concurrent condition of back pain (2 years prior and post THA) [16, 17]. Pre-operative use of opioids was identified using the same method as the post-operative use (described in the exposure of interest section). NSAIDs use was identified with ATC codes M01A* (anti-inflammatory and antirheumatic products, non-steroids).

### Statistical analysis

Descriptive statistics were used to describe the sample and OME use per 90 day post-operative period. Cox-proportional regression models were used to evaluate the risk of one and five years revision and their association with total OME amount per 90 day post-operative period. Hazard ratios (HR), 95 % confidence intervals (CI), and Wald Chi square P values are reported. Because the amount of opioid intake naturally varies during THA rehabilitation and in order to evaluate the independent effect of the amount of opioid throughout different periods of this rehabilitation, a model for each specific post-operative 90 day exposure period was created. In the first 90 days post-surgery (discharge days 1–90) the entire sample size was included in the model (*N* = 9943), in the second 90 days (days 91–180) period all survivors and non-revised cases from the first period were included (*N* = 9735), in the third 90 days (days 181–270) period all survivors and non-revised cases from the second period were included (*N* = 9638), and in fourth period (days 271–360) all survivors and non-revised cases from the third period were included (*N* = 9552). The categories of OME total amount evaluated included no opioids (reference category), low (less that the lowest quartile of the total dose in that period), medium-low (between lowest quartile and median dose), medium-high (between the median dose and third quartile dose), and high (those taking more than the highest quartile of opioids). All models were adjusted for age (continuous), gender (male vs. female), primary diagnosis for THA surgery (primary coxarthrosis vs. other), co-morbidity disease count (continuous), history of back pain (yes vs. no), prior opioid total amount per 90 day period (continuous), and NSAIDs use (yes vs. no). In the last time period studied, the number of events was small (*n* = 24) and therefore a multivariable model was not created. Proportional hazard assumptions were met (checked using time-dependent variables), collinearity was checked using tolerance values (all values >0.10), and outliers were reviewed and excluded from the sample. The alpha level chosen for statistical significant was 0.05 and tests reported are two sided. SAS 9.4 (SAS Institute, Cary, NC, USA) was used for all analyses.

### Ethics

Ethics approval was obtained from the Australian Department of Veterans’ Affairs (DVA, E010/010) and the University of South Australia (P099-10) Human Research Ethics Committees. A waiver of informed consent was obtained for this study due to the nature of the data used for this study.

## Results

There were 9943 THAs evaluated, of which 2.0 % (*N* = 200) were revised within one year and 4.2 % (*N* = 413) within five years of their index procedure. The cohort was 50.8 % (*N* = 5046) female, the median age was 81.1 years old (interquartile range (IQR) = 76.6-84.6), and 82.0 % (*N* = 8156) had a diagnosis of primary coxarthrosis. The median number of co-morbidities patients had was five (IQR 3–8) and the prevalence of back pain was 14.4 % (*N* = 1430). See Table 1.

**Table 1:** Total Hip Arthroplasty Sample Description, 2001-2012

		*Total Sample*	
		N	(%)
Total N	All	9943	100.0
Gender	Female	5046	50.8
	Male	4897	49.3
Age, years (median, IQR)		81.1	76.6-84.6
Primary Diagnosis (ICD-10-AM code and description)	M161: Other primary coxarthrosis	8156	82.0
	M169: Coxarthrosis unspecified	925	9.3
	M8795: Unspecified osteonecrosis pelvis thigh	271	2.7
	M171: Other primary gonarthrosis	152	1.5
	M160: Primary coxarthrosis bilateral	118	1.2
	Other	321	3.2
RxRisk-V number of co-morbid conditions	None	1009	10.2
	1 and 2	690	6.9
	3 and 4	1808	18.2
	5 and 6	2518	25.3
	≥7	3918	39.4
Back pain		1430	14.4
Revised within 1 Year		200	2.0
Revised within 5 Years		413	4.2

IQR=Interquartile range. ICD - 10 - AM= International Classification of Diseases, 10^th^ Revision, Australian Modification

**Fig. 1:**
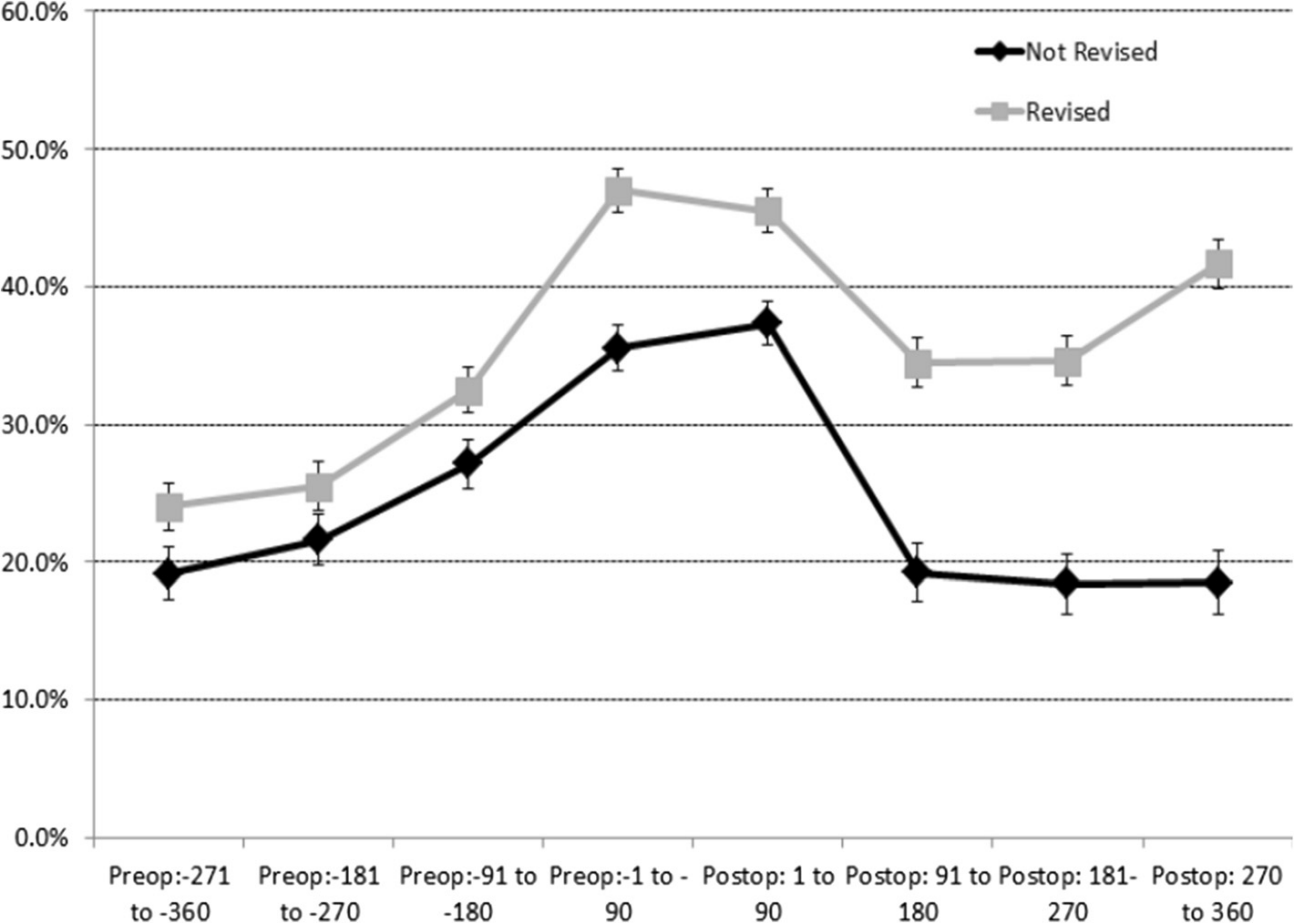
Proportion and 95 % Confidence Intervals of Patients Dispensed Opioids by 90 day Exposure Periods Pre- and Post-Total Hip Arthroplasty by 1 Year Revision Status

**Fig. 2:**
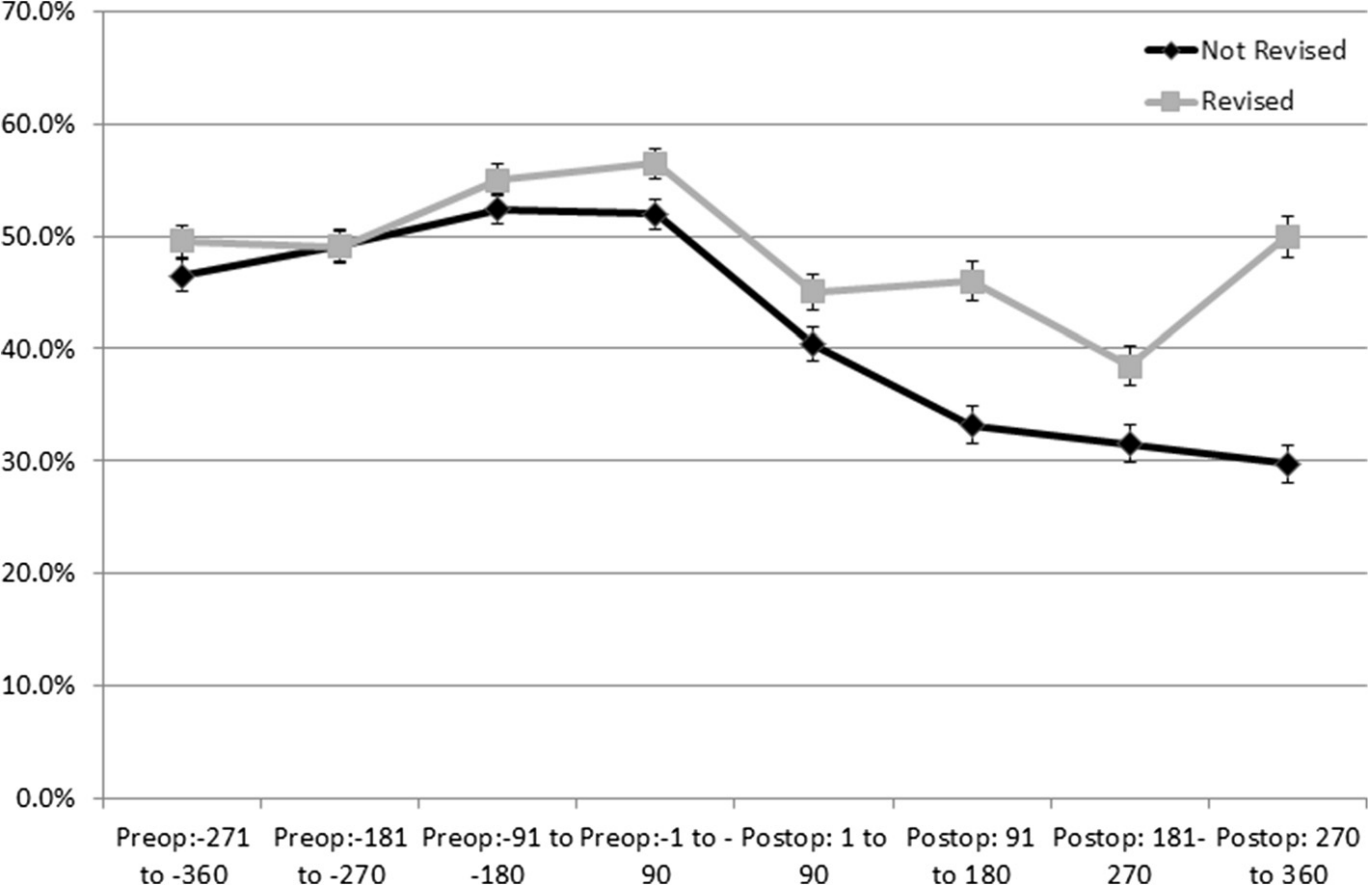
Proportion and 95 % Confidence Intervals of Patients Dispensed NSAIDs by 90 day Exposure Periods Pre- and Post-Total Hip Arthroplasty by 1 Year Revision Status

In patients who went on to be revised within one year, there was a higher proportion of pre (range 24.0 %-47.0 %) and post-operative opioids users (range 34.5 %-45.5 %), than in those not revised (pre range 19.1 %-35.5 % and post range 18.5 %-37.3 %), Fig. 1. Patients who went on to be revised within one year also had higher use of post-operative NSAID in each quarter after surgery (range 38.5 %-50.0 % compared to 29.7 %-40.3 %). Before the THA, however, the proportion of users taking NSAIDs was similar in both groups, Fig. 2. Similar proportions of opioid use were observed for cohorts of patients with five year estimates to one year estimates (data not shown).

**Fig. 3:**
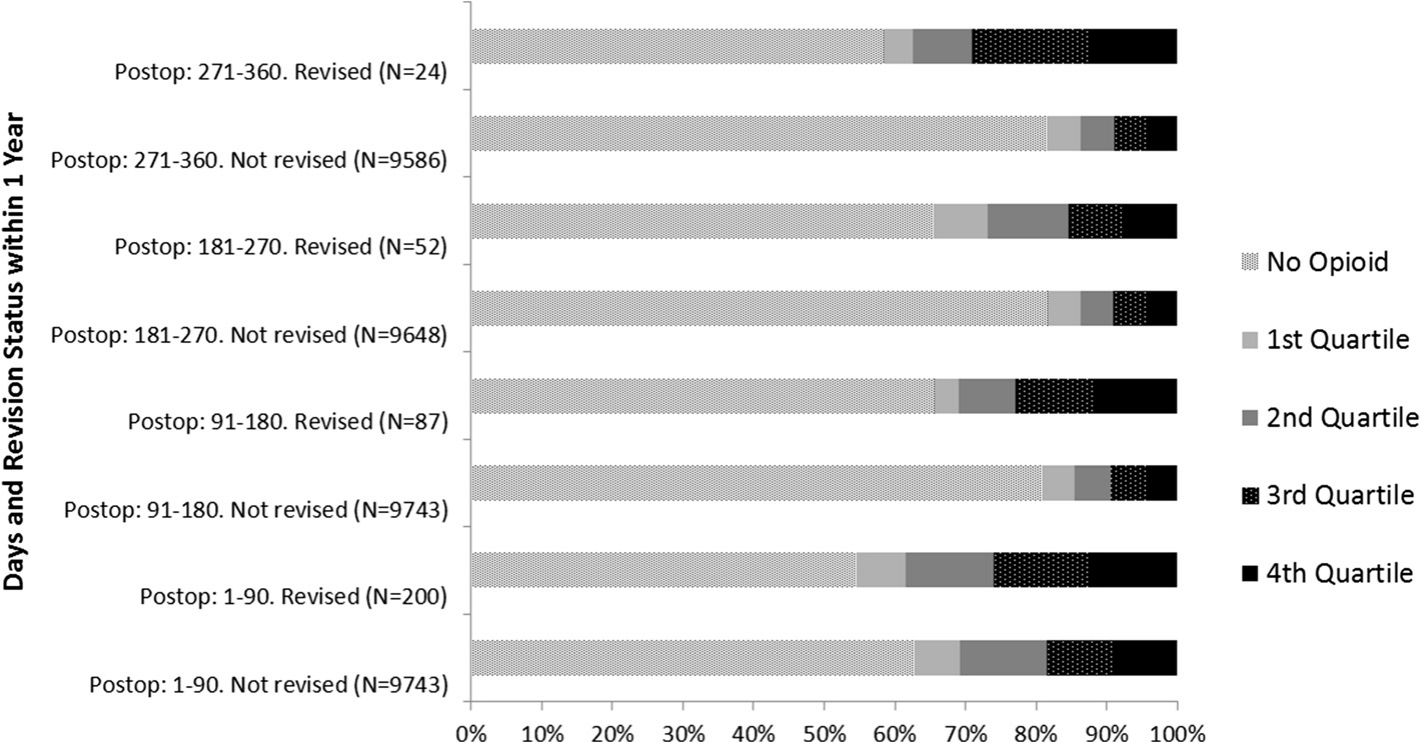
Proportion of Patients Taking Total Oral Morphine Equivalent Quartiles^1^ by 90-day Exposure Periods Post-Total Hip Arthroplasty and by 1 Year Revision Status. ^1^Quartiles amounts vary by 90 day period after surgery. See Table 2 for total amounts ranges per quartile per 90 day period

Revised patients used higher amounts of opioids (as measured by medium-high and high OMEs in each specific exposure period) than those who were not revised. See Fig. 3 for OMEs taken by the patients by 90 day periods (post THA) in patients who were revised within one year and those who were not. The categorisation by OME amounts and one and five year revision status are specified in Table 2.

**Table 2:** Total Amount of Oral Morphine Equivalents by Post - Total Hip Arthroplasty Period and by Revision Status (1 and 5 Years), 2001–2012

	*Oral Morphine Equivalent* *Amount* *(mg range)*	*Revised within 1 Year*				*Revised within 5 Years*			
		No		Yes		No		Yes	
		N	(%)	N	(%)	N	(%)	N	(%)
Days 1 - 90									
Total N		9743	98.0	200	2.0	9530	95.8	413	4.2
OME Quartiles	No Opioid	6108	62.7	109	54.5	5986	62.8	231	55.9
	Low (<150)	639	6.6	14	7.0	620	6.5	33	8.0
	Medium-low (150–349.9)	1192	12.2	25	12.5	1167	12.3	50	12.1
	Medium-high (350–839.9)	920	9.4	27	13.5	902	9.5	45	10.9
	High (≥840)	884	9.1	25	12.5	855	9.0	54	13.1
Days 91-180^a^									
Total N		9648	99.1	87	0.9	9435	96.9	300	3.1
OME Quartiles	No Opioid	7793	80.8	57	65.5	7633	80.9	217	72.3
	Low (<116.5)	453	4.7	3	3.5	438	4.6	18	6.0
	Medium-low (116.5-399.9)	484	5.0	7	8.1	476	5.1	15	5.0
	Medium-high (400–1119.9)	487	5.1	10	11.5	471	5.0	26	8.7
	High (≥1120)	431	4.5	10	11.5	417	4.4	24	8.0
Days 181-270^a^									
Total N		9586	99.5	52	0.5	9373	97.3	265	2.7
OME Quartiles	No Opioid	7822	81.6	34	65.4	7654	81.7	202	76.2
	Low (<116.5)	449	4.7	4	7.7	438	4.7	15	5.7
	Medium-low (116.5-399.9)	442	4.6	6	11.5	432	4.6	16	6.0
	Medium-high (400–1199.9)	450	4.7	4	7.7	438	4.7	16	6.0
	High (≥1200)	423	4.4	4	7.7	411	4.4	16	6.0
Days 271-360^a^									
Total N		9528	99.7	24	0.3	9315	97.5	237	2.5
OME Quartiles	No Opioid	7767	81.5	14	58.3	7607	81.7	174	73.4
	Low (<95)	456	4.8	1	4.2	444	4.8	13	5.5
	Medium-low (95–399.9)	447	4.7	2	8.3	432	4.6	17	7.2
	Medium-high (400–1216.7)	445	4.7	4	16.7	432	4.6	17	7.2
	High (≥1216.8)	413	4.3	3	12.5	400	4.3	16	6.8

OME=oral morphine equivalents

^a^Patients with revisions or who died in the previous quarter were removed from denominator. In days 91–180 207 were removed (41 were revised and died, 94 died only, and 72 were revised only). In days 181–270 97 were removed (12 were revised and died, 62 died only, 23 were revised only). In days 271–360 86 were removed (12 were revised and died, 58 died only, and 16 were revised only)

After adjustments we found that the amount of OMEs used in the first 90 days after surgery was not associated with a higher risk of revision within one or five years. In the second 90 day period after THA (days 91 to 180), patients who had the medium-high amounts of OME (OME between 400 and 1119 mg) had a 2.22 (95 % CI 1.08-4.56) times higher risk of one year revision and a 1.66 (95 % CI 1.08-2.56) times higher risk of five year revision than a patient that was not taking any opioids. During the same period, patients taking the highest amounts of OMEs (OME ≥1120 mg) had a 2.64 (95 % CI 1.03-6.74) times higher risk of one year revision and a 2.11 (95 % CI 1.13-3.96) times higher risk of five year revision. In the third 90 days post-operative period (days 181 to 270) there was not an association between the levels of OMEs and revision. Finally, in the last period evaluated (days 271–360) there was a higher crude risk of one year revision in patients in the medium-high and high levels of OME but we could not adjust for other variables due to the small number of events (*n* = 24). For the same time period, there was not an adjusted higher risk of five years revision. See Table 3.

**Table 3:** Crude and Adjusted Risk of 1 Year and 5 Year Revisions by Total Amount of Oral Morphine Equivalent (mg) Quartiles by 90 day Periods Post- Total Hip Arthroplasty

		1 Year Revision			5 Years Revision		
	*OMEs (mg range), Reference=No opioid*	*Crude HR*	*Adjusted HR*	*P Value*	*Crude HR*	*Adjusted HR*	*P Value*
Days 1 - 90^a^	Low (<150)	1.22 (0.70-2.12)	1.12 (0.64-1.96)	0.696	1.39 (0.96-2.00)	1.29 (0.89-1.86)	0.177
	Medium-low (150–349.9)	1.16 (0.75-1.80)	1.01 (0.65-1.56)	0.973	1.16 (0.86-1.58)	1.06 (0.78-1.44)	0.715
	Medium-high (350–839.9)	1.62 (1.07-2.47)	1.32 (0.86-2.04)	0.209	1.34 (0.97-1.85)	1.14 (0.82-1.59)	0.422
	High (≥840)	1.56 (1.01-2.40)	1.02 (0.60-1.76)	0.943	1.69 (1.26-2.27)	1.24 (0.85-1.81)	0.269
Days 91 - 180^a^	Low (<116.5)	0.91 (0.28-2.90)	0.77 (0.24-2.49)	0.667	1.48 (0.92-2.40)	1.35 (0.83-2.18)	0.230
	Medium-low (116.5-399.9)	1.98 (0.90-4.34)	1.64 (0.74-3.66)	0.223	1.15 (0.68-1.94)	0.99 (0.58-1.69)	0.982
	Medium-high (400–1119.9)	2.80 (1.43-5.49)	2.22 (1.08-4.56)	0.030	1.98 (1.32-2.97)	1.66 (1.08-2.56)	0.022
	High (≥1120)	3.14 (1.60-6.15)	2.64 (1.03-6.74)	0.042	2.15 (1.41-3.27)	2.11 (1.13-3.96)	0.020
Days 181 - 270^b^	Low (<116.5)	2.05 (0.73-5.77)	1.70 (0.60-4.84)	0.321	1.34 (0.80-2.27)	1.21 (0.72-2.06)	0.472
	Medium-low (116.5-399.9)	3.11 (1.31-7.41)	2.42 (1.00-5.87)	0.050	1.48 (0.89-2.46)	1.25 (0.75-2.10)	0.396
	Medium-high (400–1199.9)	2.05 (0.73-5.78)	1.28 (0.43-3.85)	0.659	1.44 (0.87-2.39)	1.01 (0.59-1.73)	0.977
	High (≥1200)	2.16 (0.77-6.10)	0.48 (0.08-3.01)	0.432	1.60 (0.96-2.67)	0.65 (0.27-1.60)	0.349
Days 271 - 360^c^	Low (<95)	1.22 (0.16-9.31)	NA	NA	1.35 (0.77-2.36)	1.19 (0.67-2.10)	0.238
	Medium-low (95–399.9)	2.47 (0.56-10.88)	NA	NA	1.79 (1.09-2.95)	1.55 (0.94-2.58)	0.088
	Medium-high (400–1216.7)	4.96 (1.63-15.07)	NA	NA	1.81 (1.10-2.97)	1.41 (0.83-2.38)	0.203
	High (≥1216.8)	4.01 (1.15-13.96)	NA	NA	1.93 (1.16-3.23)	1.22 (0.55-2.73)	0.627

OME=oral morphine equivalents. NA= Not applicable

^a^Models adjusted for age, gender, primary diagnosis, RxRisk-V number of co-morbidities, back pain, prior opioid use, and NSAID use

^b^1 year revision model adjusted for prior opioid use, back pain and RxRisk. 5 year revision model adjusted for age, gender, primary diagnosis, RxRisk-V number of co-morbidities, back pain, prior opioid use, and NSAID use

^c^1 year revision estimates could not be adjusted because of the small number of events in this period (*N*=24). 5 year revision model adjusted for age, gender, primary diagnosis, RxRisk-V number of co-morbidities, back pain, prior opioid use, and NSAID use

## Discussion

In this elderly cohort of THA patients, we estimated the proportion of patients still taking pain relief at days 271 to 360 post-surgery to be between 19 % and 42 %, depending on whether they went on to be revised or not. These were the proportion of patients who were still taking some opioid medications during this period, a period which is no longer considered surgical recovery. Our results are consistent with previous estimates that persistent post-operative pain occurs in 7 % to 23 % of primary THA patients [3]. When we investigated opioid use, we found higher amounts of opioid medication taken after THA to be associated with higher risk of revision. Specifically, patients taking 400 mg-1120 mg and ≥1120 mg total amounts of OMEs during days 91 to 180 post-operative, or 4-12 mg per day, had a higher likelihood of revision surgery within one year and five years.

Patient reported post-operative pain is an important clinical THA outcome. In addition to its value in clinically assessing the procedure, it is also associated with other hard endpoints, such as revision surgery [18]. Britton et al. reported that patients with severe or moderate pain after surgery were more likely to have revisions than patients with stiffness [18]. Of the patients in their study with severe or moderate pain post-operatively (*n* = 351 out of 2268), 28 % (*n* = 100) had a revision within 2 years and 33 % (*n* = 115) within 5 years. In our cohort, 42 % of patients who went on to be revised were being treated for pain with opioids at one year post-operatively, a similar number of patients compared to Britton et al.’s study. In our study we also found the amount of opioid use, and not just whether opioids were being used, in the days 91–180, to be associated with a higher risk of revision. The prevalence of opioid use and amount of use also continued to be higher in patients with revisions in the third and fourth quarter post-operative suggesting a consistent trend but the actual risk of revision was not statistically significantly different, which could be due to the smaller number of events in these models or possibly that the later use of opioids is not as helpful as an indicator for revision.

Only one study, to our knowledge, reported both the prevalence of pain (8.1 %) and opioid use (2.3 %) at two years post-operatively. This study did not evaluate any association of these indicators with revision surgery [19]. The prevalence of pain, measured either by the patient reported instruments or opioid use, reported by Singh et al.’s study is significantly lower than both Britton et al.’s and our study. This is possibly due to the assessment being based on patients’ reported estimates rather than pharmaceutical records, the former which could suffer from response bias [19]. We are not aware of studies that have included opioid use as a proxy for pain prevalence after joint arthroplasty and therefore no direct comparison to our observations can be made. Additionally, no comparisons to other literature can be made to our observations of a higher risk of revision and total amount of opioid use.

The use of opioid medication in our cohort was significantly higher in the revision group before their primary procedure. This observation agrees with reports of other surgical procedures that the degree of pre-operative pain is associated with post-operative pain [20]. The use of opioids pre-operatively is associated with poorer clinical outcomes in THA patients [21]. However, a higher utilisation of opioids before and after surgery in patients who go on to require revision has not been previously shown. It is worth noting that our analyses were adjusted for other analgesic use as well in an attempt to evaluate the independent effect of opioid at each time period with the risk of revision (i.e. remove any confounding introduced by other analgesic use).

Surrogate measures for the areas evaluated by patient reported outcome tools, such as analgesic uptake for pain management, are viable alternatives to traditional ascertainment of patient reported outcomes. Prescription medication claims data are available in certain countries, and with proper understanding of the limitations involved with these data, they are a possible source for monitoring signals of potential poorer outcomes [22–25]. In this study, we were able to obtain the patients’ prescription dispensing and evaluate whether the levels of opioid intake during the study period were associated with the risk of revision surgery. Dispensing of opioids seems to be a viable alternative for capturing patient’s pain within 91–180 days post-operative and offers an opportunity to identify patients at high risk of revision surgery.

Our study has several limitations. The data used are administrative and subjective to potential coding errors and under-reporting. However, the limitations of our data are not likely to be different between the groups studied and we, therefore, expect this to have minimal impact on our estimations. The fact that medication can be prescribed for many reasons and we do not know whether the prescriptions obtained by our cohort were for the joint studied is another limitations. Because of this, we controlled our analysis for other reasons why these patients may have received these medications. We also restricted patients to include only those with one joint procedure within one year. It is possible residual confounding, due to other unknown or unavailable data, is present. Our findings may also have limited generalisability because we have an older cohort of patients. Another limitation includes our inability to account for different opioid prescribing practices and preferences of physicians and surgeons with the available data, which could confound the reported findings. Further, there are also few revisions occurring during days 180–360 and it is possible that we were underpowered to estimate the risk of one year revision for the number of opioid exposures chosen for the study, however, the estimates were similar to the 5 year risk estimates, which had models with a substantial greater number of events. And while we chose a method of opioid exposure classification that accounted for both the time of exposure (i.e. each 90 day period) and the total amount of opioid use during these times, we recognize that there are multiple ways to evaluate opioid exposure, such as binary exposure to the medication, average daily use, cumulative amount over time, and this should be recognized when interpreting our results and comparing to other studies on opioid use in joint surgery. Our study is also an exploratory analysis of the association of opioid amounts and the risk of revision surgery in the short and mid-term, we did not conduct multiple hypothesis adjustments to our analysis but would recommend it for subsequent confirmatory analysis. Finally, while we used published methodology to identify revision cases, it is possible the revision surgery was linked to incorrect primary procedures due to the lack of laterality in our data [26, 27]. We do not believe this to be different between the groups studied and do not expect this to impact our estimations.

Our study strengths include the captive DVA population and comprehensive claims data available for these patients’ services, which we used in this study to obtain patients’ episodes of care as well as their prescription information. Additionally, there is no attrition, apart from death, in this cohort, so all events are captured. Finally, because of the type of data used to identify opioid use our estimations do not suffer from patient response bias- either from not filling out a questionnaire or not filling it out properly due to lack of understanding of questions [28].

## Conclusion

In this cohort of older THA patients, opioid medication in the year prior to and after surgery was 19 % to 42 % and varied between patients who had a revision surgery or not. Patients using moderate to high amounts of opioid, during days 91–180 post surgery had a higher risk of revision within one and five years than patients not taking opioids. The observation that patients with revision procedures always had higher opioid consumption than patients that did not go on to be revised, including in the pre-operative period, offers a window of opportunity for surgeons to address opioid use and its possible post-operative impact pre surgery, when more frequent contact and surgical counselling is occurring. Additionally, identifying that higher amounts of opioids post-surgery indicate a higher risk of revision suggests that opioid use, specifically within 91–180 days, is a proxy for pain and a useful indicator for revision and should be closely monitored by healthcare providers.

## Abbreviations

THA: Total Hip Arthroplasty
OME: Oral Morphine Equivalent
NSAID: non-steroidal anti-inflammatory drug
DVA: Department of Veterans’ Affairs
ATC: Anatomic, Therapeutic and Chemical classification
PBS: Pharmaceutical Benefits Schedule
ICD-10-AM: International Classification of Disease, 10^th^ Revision, Australian Modification
HR: Hazard Ratio
CI: Confidence Intervals
IRQ: Interquartile Ranges
